# Shell–Core Microbeads Loaded with Probiotics: Influence of Lipid Melting Point on Probiotic Activity

**DOI:** 10.3390/foods13142259

**Published:** 2024-07-17

**Authors:** Youfa Xie, Kui Zhang, Jingyao Zhu, Li Ma, Liqiang Zou, Wei Liu

**Affiliations:** 1 State Key Laboratory of Food Science and Resources, Nanchang University, Nanchang 330047, China; xieyoufa@crjz.com (Y.X.); zk244833@163.com (K.Z.); ziy1262000@163.com (J.Z.); 18772306736@163.com (L.M.); zouliqiang2010@163.com (L.Z.); 2 Jiangzhong Pharmaceutical Co., Ltd., Nanchang 330041, China; 3 International Institute of Food Innovation Co., Ltd., Nanchang University, Luozhu Road, Xiaolan Economic and Technological Development Zone, Nanchang 330200, China; 4 National R&D Center for Freshwater Fish Processing, Jiangxi Normal University, Nanchang 330022, China

**Keywords:** shell–core microbead, probiotic, high-precision concentric drop formation technology, lipid melting point, probiotic viability

## Abstract

Probiotics have many beneficial physiological activities, but the poor stability during storage and gastrointestinal digestion limits their application. Therefore, in this study, a novel type of shell–core microbead for loading probiotics was prepared through high-precision concentric drop formation technology using gelatin as the shell material and lipids as the core material. The microbeads have a regular spherical structure, uniform size, low moisture content (<4%) and high probiotic activity (>9.0 log CFU/g). Textural testing showed that the hardness of the medium-chain triglyceride microbeads (MCTBs), cocoa butter replacer microbeads (CBRBs) and hydrogenated palm oil microbeads (HPOBs) increased gradually (319.65, 623.54, 711.41 g), but their springiness decreased (67.7, 43.3, 34.0%). Importantly, lipids with higher melting points contributed to the enhanced stability of probiotics during simulated digestion and storage. The viable probiotic counts of the HCTBs, CBRBs and HPOBs after being stored at 25 °C for 12 months were 8.01, 8.44, and 8.51 log CFU/g, respectively. In the simulated in vitro digestion process, the HPOBs resisted the destructive effects of digestive enzymes and gastric acid on probiotics, with a reduction in the probiotic viability of less than 1.5 log CFU/g. This study can provide new ideas for the preparation of intestinal delivery probiotic foods.

## 1. Introduction

Probiotics are living microorganisms that, when administered in adequate amounts, confer health benefits to the host [1]. Their benefits include boosting immunity [2], preventing diarrhea [3], preventing colon diseases [4], and others. The dosage of probiotics is reported to be a critical factor determining their effects on human health, with food products needing to contain at least 6.0 log CFU/g of viable probiotics to ensure sufficient bacteria for their beneficial effects in the body [5,6]. However, external factors such as water, high temperature, light, and oxygen can affect the viability of probiotics in food matrices, making it challenging to maintain their stability during processing and storage [7]. Additionally, after ingestion, probiotics are significantly inactivated by the highly acidic environment of the stomach and the high bile salt concentrations in the small intestine, making it difficult to maintain a sufficient count of viable probiotics to exert their beneficial effects [8]. Therefore, in order to reduce the inactivation of probiotics and preserve their viability, research on constructing probiotic delivery systems has become increasingly popular.

Several methodologies have been developed to safeguard probiotics, including spray-drying [9], freeze-drying [10], emulsification [11], hydrogelation [12], electrospinning [13], etc. Spray-drying is popular in the microbiological industry to produce powdered probiotics due to its high production efficiency and low cost. However, the high temperature, osmosis and oxidative stress during spray-drying can significantly deactivate probiotics [14]. Compared to spray-drying, the freeze-drying process is gentler and results in higher probiotic survival rates, but its slow drying speed and high cost limit its application [10]. Emulsification and hydrogelation are also frequently employed for probiotic encapsulation to improve the gastrointestinal stability of probiotics. Xiao et al. used the hydrogel made from thiolated hyaluronic acid to encapsulate *Lactobacillus rhamnosus* ATCC 7469, enhancing their viability against gastrointestinal tract insults [15]. Silva et al. applied an emulsification method to encapsulate *Lactobacillus plantarum* and achieved good results [11]. However, these encapsulation systems usually have a high moisture content, which hinders the long-term storage stability of probiotics. Additionally, electrospinning is currently employed for the encapsulation of probiotics. The electrospinning process conditions are relatively mild, and probiotics encapsulated within electrospun nanofibers exhibit improved stability and functionality. But due to the frequent introduction of organic solvents in the electrospinning process, potential safety issues may arise [16].

The use of lipids or oil gels for probiotic encapsulation to enhance their stability has been established as a reliable strategy [17,18]. According to Picot and Lacroix, lipids offer protective properties to microorganisms by impeding the diffusion of hydrogen ions, organic acids, moisture, and oxygen through the lipid membrane [19]. Klindt-Toldam et al. observed a negligible reduction in probiotic viability during the encapsulation of *Lactobacillus acidophilus* NCFM and *Bifidobacterium lactis* HN019 in chocolate, with the viable cell counts remaining relatively stable after storage for 12 months at 4 °C or 18 °C [20]. Silva et al. utilized semi-sweet chocolate as a carrier for the probiotic strains *Lactobacillus acidophilus* LA3 and *Bifidobacterium animalis* subsp. *lactis* BLC1, reporting minimal reductions of 1.4 log CFU/g and 0.7 log CFU/g, respectively, in the probiotic populations within the chocolate after storage at 25 °C for 120 days [18]. Pedroso et al. encapsulated live *Lactobacillus acidophilus* and *Lactobacillus casei* in hydrogenated palm oil, resulting in the improved storage and digestive stability of the probiotics [21]. Additionally, Azeem et al. demonstrated the significant potential of lipids in extending probiotic viability under adverse conditions [22]. However, the exclusive reliance on lipid encapsulation for probiotics is constrained by factors such as the susceptibility to oxidation, perceptible greasiness, and challenges in storage.

Shell–core structure capsules represent a more promising carrier for encapsulating probiotics compared to pure lipids. It not only places probiotics in isolated lipid environments to enhance their stability but also offers advantages such as controlled release, isolation from the external environment, easier processing and storage, and masking of core stimulating flavors [23,24]. This structure is also commonly used for encapsulating lipid-soluble active ingredients or hydrophilic components sensitive to moisture [25], such as plant seed extracts, vitamins, minerals, medicines, etc. Chen et al. investigated the encapsulation of corn zein with an outer layer composed of algae oil and vanillin to fabricate microcapsules [26]. Their study revealed that manipulation of the shell thickness effectively retarded the oxidation of algae oil and facilitated controlled release of volatile components. This finding aligns with previous research indicating that shell–core structured capsules exhibit a capacity to decelerate the degradation of food constituents and mask undesirable sensory attributes [27].

Therefore, this study reports a novel type of probiotic microbead with a shell–core structure prepared using high-precision concentric droplet formation technology. Gelatin was chosen as the shell material due to its excellent film-forming properties, gas barrier characteristics, biodegradability, and relatively low cost [28], while probiotics are encapsulated in an internal lipid core to avoid contact with moisture and oxygen. This study also investigated the effects of three different lipids with varying melting points: medium-chain triglyceride (MCT), cocoa butter replacer (CBR), and hydrogenated palm oil (HPO) as core materials on the physicochemical properties of the microbeads. Additionally, this study examined the influence of these lipids on the processing, in vitro digestion, and the viability during storage at both 4 °C and 25 °C of probiotics. The research findings hold significant potential for a wide range of applications and can provide guidance and theoretical foundations for the development of products related to active probiotics.

## 2. Materials and Methods

### 2.1. Materials

*Lactobacillus plantarum* P9 was obtained from Jiangzhong Pharmaceutical Co., Ltd. (Nanchang, China). Medium-chain triglyceride (MCT) was purchased from Qingdao Haizhiyuan Biotechnology Co., Ltd. (Qingdao, China), cocoa butter replacer (CBR, melting point 35.6 °C) was purchased from Yihai Kerry Arawana Holdings Co., Ltd. (Shanghai, China), and hydrogenated palm oil (HPO, melting point 41.2 °C) was purchased from Yihai Kerry Arawana Holdings Co., Ltd. (Shanghai, China). MRS AGAR culture medium was purchased from Beijing Solarbio Science and Technology Co., Ltd. (Beijing, China). Gelatin (from pig skin, 280 Bloom) was purchased from Jiangxi Fumeitai Biotechnology Co., Ltd. (Jiujiang, China). Glycerin was purchased from KLK Company (Kuala Lumpur, Malaysia). The Live/Dead^®^ BacLight™ bacterial activity kit was purchased from Thermo Fisher Scientific (Waltham, MA, USA) and all the other reagents were of analytical grade.

### 2.2. Preparation of Probiotic Shell–Core Microbead

#### 2.2.1. Configuration of Shell Materials

Add gelatin (15%, *w*/*w*) and glycerol (1%, *w*/*w*) into distilled water, and stir in a water bath (HH-4, Jiangsu Jintan Xiaoyang Electronic Instrument Factory, Jintan, China) at 55 °C until the solution is clear and transparent.

#### 2.2.2. Configuration of Core Materials

Configuration of medium-chain triglyceride core material: Add 1% (*w*/*w*) of *Lactobacillus plantarum* P9 powder to MCT. Use a disperser (T18 Brushless digital disperser, Germany IKA company, Staufen im Breisgau, Germany) at 5000 rpm for 2 min to disperse it uniformly, forming a homogeneous liquid core phase.

Configuration of cocoa butter replacer core material: Heat cocoa butter with a melting point of 35.6 °C until melted. When cooled to 40 °C, add 1% (*w*/*w*) of *Lactobacillus plantarum* P9 powder and disperse uniformly. Keep at 45 °C for later use.

Configuration of hydrogenated palm oil core material: Heat hydrogenated palm oil with a melting point of 41.2 °C until melted. When cooled to 45 °C, add 1% (*w*/*w*) of *Lactobacillus plantarum* P9 powder and disperse uniformly. Keep at 45 °C for later use.

#### 2.2.3. Preparation of Probiotic Shell–Core Microbead

The shell–core microbead preparation method follows the research of Gao et al. [23]. The shell material and the core material are poured into the shell material tank and the core material tank of the concentric microbead equipment (DWJ-2000-JW-2T, Shandong Baiyao Tai Traditional Chinese Medicine Technology Co., Ltd., Jinan, China), respectively, where the core material tank is continuously stirred at a low speed to ensure the even distribution of the probiotics. Adjust the machine parameters: the temperature of the shell material tank is set to 50~55 °C, the core material tank temperature is 45 °C, and the temperature of the cooling circulating fluid is 7~9 °C. The flow rate ratio of the shell material to core material is 1:3. The wet microbeads produced are dried in a room with a temperature of 25~30 °C and a relative humidity of 30~40% for 10–12 h to obtain the finished product. Using the aforementioned method, probiotic microbead samples of three different melting point lipid matrices, namely medium-chain triglyceride, cocoa butter substitute, and hydrogenated palm oil, are prepared and named as medium-chain triglyceride microbeads (MCTBs, negative control), cocoa butter replacer microbeads (CBRBs, positive control), and hydrogenated palm oil microbeads (HPOBs, positive control), respectively.

### 2.3. Basic Properties of Microbeads

Evaluate the basic properties of the microbeads. Observe and photograph the changes in the appearance of the microbead samples with different core materials before and after drying. Measure the diameter of the microbeads using a vernier caliper. The weight of the microbeads can be obtained by the analytical balance. The method of Yavari et al. can be slightly modified to test the moisture content of the microbeads [29], where the microbeads are placed in an oven at 105 °C and weighed every 2 h until a constant weight is achieved, at which point the moisture content of the microbeads is calculated. Use the texture analyzer to measure the influence of different melting point lipids as cores on the hardness and springiness of the microbeads, according to the method of Cao et al., with slight modifications. The texture analyzer (TA-XT-Plus, Stable Microsystems, Godalming, UK) and a P/0.5R cylindrical probe are used to measure the hardness and springiness of each bead [30]. Test parameters: The pre-test speed is 1.5 mm/s, the test speed is 0.5 mm/s, the post-test speed is 0.5 mm/s, the bead strain is 20%, and the trigger force is set to 2.0 g.

### 2.4. Viability of Probiotics during Preparation

During the preparation process, the probiotics in the core material may lose their activity due to factors such as high temperature, mechanical stress, exposure to oxygen, or dehydration. To investigate the protective effects of lipids with different melting points on probiotics during the microbead manufacturing process, samples are taken from the core fluid before the microbeads’ formation, from the freshly formed microbeads, and from the completely dried microbeads to test the viability of the probiotics. The testing method, based on Danica et al., with modifications, involves shaking and mixing the samples with 5% Tween 80 physiological saline, gradient dilution, and inoculation on MRS agar culture medium. The total colony count is calculated after anaerobic cultivation at 37 °C for 48 h (YQX-T anaerobic incubator, Shel Lab Inc., Cornelius, OR, USA). All the diluent and consumables used in the culture are sterilized at 121 °C for 20 min in advance.

### 2.5. In Vitro Simulated Digestion

The method of Gao et al. is adapted and modified to test the changes in probiotic viability at each stage of the simulated in vitro digestion in microbeads with different lipid cores [31].

Oral digestion: Sodium chloride (1.594 g/L), ammonium nitrate (0.328 g/L), potassium chloride (0.202 g/L), potassium citrate (0.308 g/L), potassium dihydrogen phosphate (0.636 g/L), sodium dihydrogen urate (0.021 g/L), urea (0.198 g/L), sodium lactate (0.146 g/L), and mucin (3 g/L) are each added to deionized water according to the required amounts and stirred until completely dissolved. Then, 1.5 g of different core material mouth incense pills are mixed with 13.5 mL of simulated salivary fluid (SSF), adjusted to pH 6.8, and digested at 37 °C in a shaking water bath at 100 r/min for 10 min before sampling for bacterial activity.

Gastric digestion: NaCl and HCl are added to distilled water in the ratios of 2 mg/mL and 7 mg/L HCl, respectively. Then, pepsin is added at a ratio of 3.2 mg/mL to prepare simulated gastric fluid (SGF). After simulated oral digestion, 15 mL of simulated gastric fluid is added to the digestion bottle, adjusting the pH to 2.0, and incubated with shaking at 100 r/min for 2 h before sampling for bacterial activity.

Intestinal digestion: CaCl_2_ and NaCl are dissolved in distilled water in the ratios of 36.7 mg/mL and 218.7 mg/mL, respectively, to prepare simulated intestinal fluid (SIF). Adjust the pH of the mixture after gastric digestion to 7.0. Then, 2.5 mL of lipase, 2.5 mL of pancreatin and 3.5 mL of bile salt (digestive enzymes are dissolved in 5 mM PBS at pH 7.0) and 1.5 mL of simulated intestinal fluid are added.

### 2.6. Fluorescence Characteristics of Probiotics

The Live/Dead^®^ BacLight™ bacterial activity kit commercial assay (Thermo Fisher Scientific, MA, USA) is used to stain the probiotics. Probiotics with intact cell membranes emit green fluorescence, while probiotics with damaged cell membranes emit red fluorescence. The excitation wavelength for SYTO 9 dye is 485 nm, with an emission wavelength of 498 nm. The excitation wavelength for PI dye is 535 nm, with an emission wavelength of 617 nm. The probiotics are fluorescently labeled using a fluorescence microscope (DM18, Leica Microsystems CMS Gmbh, Wetzlar, Germany) with a 20× objective [32].

### 2.7. Storage Viability

The storage conditions may potentially affect the survival ability of probiotics due to various environmental factors [33]. The microbead samples are placed at 4 °C and 25° C, respectively. Every three months, samples are taken from each environment to measure the viable bacterial count. The obtained data are then compared.

### 2.8. Statistical Analysis

Statistical analysis is conducted using Origin2017, with each experiment repeated three times. The differences between groups are analyzed using SPSS 26 software, and the significance level is set at *p* < 0.05.

## 3. Results and Discussion

### 3.1. The Basic Properties of Microbeads

**Figure 1:**
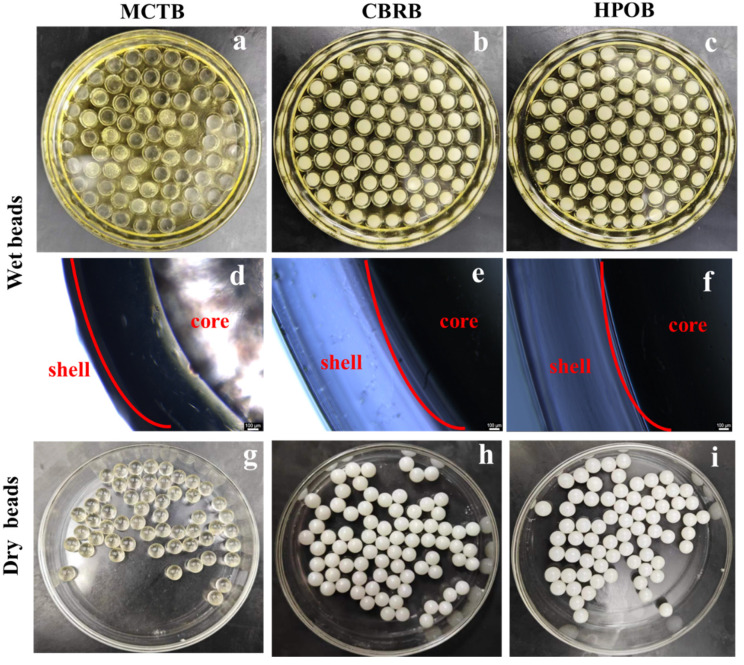
Apparent images of microbeads: MCTBs before drying (**a**,**d**) and after drying (**g**), CBRBs before drying (**b**,**e**) and after drying (**h**), HPOBs before drying (**c**,**f**) and after drying (**i**).

The microbeads prepared using high-precision concentric drop formation technology with a shell–core structure contribute to improving the stability of probiotics. Different microbeads prepared from various lipid matrices are shown in Figure 1. In Figure 1a–f, images of the MCTB, CBRB, and HPOB probiotic beads before drying are shown, both in apparent and microscopic (5×) views. It can be observed that all the microbeads exhibit a distinct shell–core structure, where the gelatin outer shell provides mechanical strength to prevent microbead breakage during subsequent processing, while the inner lipid layer helps to isolate oxygen and moisture. Figure 1g–i represent the microbeads after drying, where they appear as regular spherical shapes with consistent sizes, without any signs of concavity, rupture, protrusion, or leakage of the inner core. The weight of all three types of microbeads is approximately 110 mg, with a diameter of 6 mm. This allows them to load high amounts of probiotics while also avoiding the swallowing difficulties associated with the oversized particles (>1 cm) of traditional soft capsules, indicating the product’s potential for application in functional foods. In addition, the microbeads exhibit very low moisture content (<4%), indicating excellent microbial stability.

The hardness of the MCTBs, CBRBs, and HPOBs is 319.65 ± 35.43 g, 623.54 ± 77.73 g, and 711.41 ± 49.57 g (Table 1), respectively. The microbeads with solid lipid cores demonstrate significantly higher hardness compared to those with MCTBs, possibly due to the formation of densely packed crystal structures facilitated by solid lipids [34]. There is no statistically significant difference in hardness between the CBRBs and HPOBs (*p* > 0.05), indicating that hardness does not strictly correlate with lipid melting point elevation. This discrepancy may be attributed to factors such as crystallization behavior variations within the system [35]. On the contrary, microbeads with liquid lipid cores exhibit superior springiness compared to those with solid lipid cores. This could be attributed to the easy recovery of the microspheres’ original shape when compressed due to the presence of flowable lipid as the core, while solid-state core lipids make it difficult for microspheres to recover after compression.

### 3.2. Probiotic Activity during Preparation

The entire preparation process for the microbeads is divided into two stages. First is the microbeads’ formation stage: The shell material gelatin solution and the core lipid are transported to the concentric circular nozzle through the action of a pump to form a shell–core structure, and then the shell is rapidly cooled and shaped into a gel by the cooling circulating liquid. Secondly, the drying stage: The microbeads are placed in a rotary cage dryer and dried for 10–12 h in a room with a temperature of 25–30 °C and a relative humidity of 20–30%. Throughout the process, the probiotics may be inactivated due to the mechanical action, the high temperature when contacting the gelatin solution (50–55 °C), or the dehydration stress during drying. To investigate the viability of the probiotics throughout the entire preparation process, samples were taken and tested at each stage.

**Figure 2:**
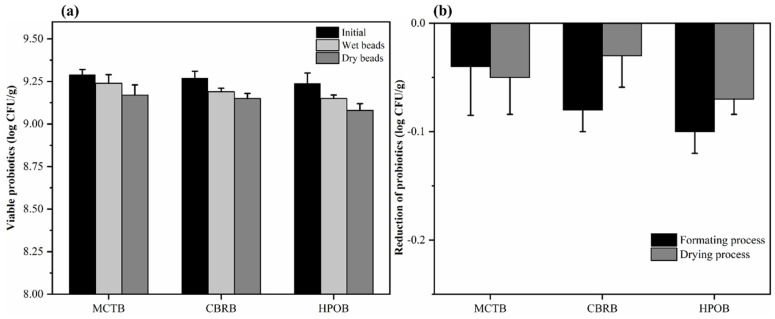
The viable counts (**a**) and reduction (**b**) of probiotics in microbeads during the microbeads’ formation and drying processes.

The changes in the viability of the bacteria in the three types of microbeads during the microbeads’ formation stage are shown in Figure 2. Initially, the viability of the probiotics in the insulated storage tank loaded with lipids was tested, with all the probiotics counting at about 9.3 log CFU/g. After the microbeads’ formation stage, the viability of the three types of microbeads was 9.24 ± 0.05 log CFU/g (MCTB), 9.19 ± 0.02 log CFU/g (CBRB), and 9.15 ± 0.02 log CFU/g (HPOB), respectively. There was no significant difference in the count of probiotics in the microbeads prepared with the three types of cores, and the reduction in the probiotic counts was less than 0.2 log CFU/g, indicating that the microbeads’ formation stage caused minimal damage to the probiotics. This is attributed to the mild temperatures used during the microbeads’ formation stage. The drying process of the microbeads also caused minimal damage to the probiotics, with the viability after drying being 9.17 ± 0.06 log CFU/g, 9.15 ± 0.03 log CFU/g, and 9.09 ± 0.04 log CFU/g, respectively. Compared to the situation before drying, there was no significant decrease in the bacterial counts (*p* > 0.05). This is because the temperature during the microbeads’ drying is relatively low (25–30 °C), which is below the level that would cause dehydration-induced inactivation of bacteria or denaturation of cellular components [36]. Additionally, the matrix that carries the probiotics is also an important factor affecting the survival rate of probiotics during drying [37]. The microbeads have a high fat content, which enables them to avoid a reduction in the count of probiotics due to exposure to moisture or oxygen during the drying process.

In conclusion, regardless of the type of lipid used as the core, the viable cell counts of the microbeads were over 9.0 log CFU/g, which is due to the mildness of the entire processing procedure and also indicates that the high-precision concentric drop formation technology is a potential method for preparing probiotic foods.

### 3.3. Viability of Probiotics In Vitro Digestion

**Figure 3:**
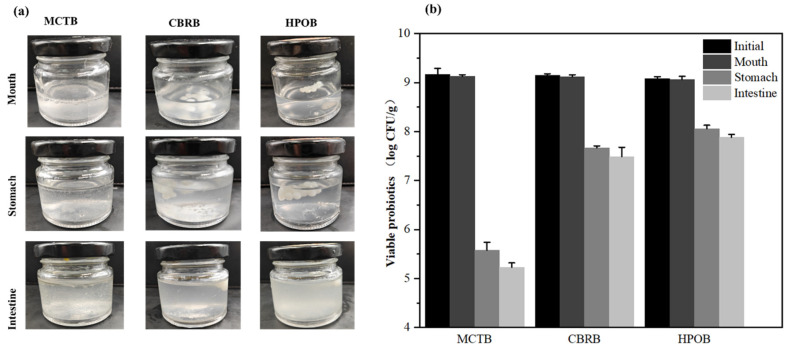
Apparent images of microbeads (**a**) and viable counts of probiotics (**b**) during in vitro digestion.

The health benefits of probiotics depend on the vitality and a sufficient quantity of the probiotics in the target intestines. To colonize the colon, probiotics must withstand harsh conditions as they pass through the upper gastrointestinal tract, where they must resist the harmful effects of acids and bile salts in the gastrointestinal tract [38]. In order to explore the changes in appearance and vitality of the probiotics in the microbeads during digestion, this study recorded apparent images at each stage of the simulated *in vitro* digestion and measured the count of probiotics, with the results shown in Figure 3.

The digestion process in Figure 3a shows that during oral digestion, due to the temperature being close to body temperature (37 °C), the triple helix structure of the gelatin gel transforms into single strands [39], the shell layer completely dissolves, and the core lipids are exposed to the digestive fluid. The core lipids released from MCTBs float on the digestive fluid, while CBRBs and HPOBs, due to the higher melting points of the core lipids, clump together during digestion and do not show significant melting. In particular, HPOBs can largely maintain a spherical structure, and the same phenomenon is observed during gastric digestion. However, during simulated intestinal digestion, due to the action of lipase, the lipids are digested, making the digestive fluid turbid.

**Figure 4:**
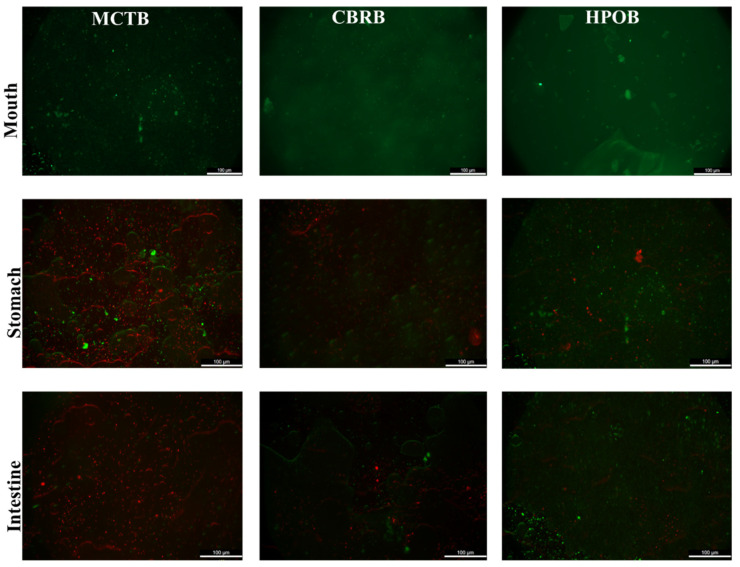
Fluorescent images of probiotics during in vitro digestion.

The viability measurement of probiotics in Figure 3b shows that there was no significant difference in the count of viable cells of the three types of microbeads after simulated oral digestion compared to before digestion (*p* > 0.05), indicating that the short duration and neutral pH conditions in the simulated salivary fluid do not affect the viable cell count of probiotics. Camelo-Silva et al. reported similar findings, where *Bifidobacterium* BB-12 added to ice cream did not show a significant reduction in the viable cell count after simulated oral digestion [40]. However, simulated gastrointestinal digestion had a significant effect on the vitality of probiotics due to the low pH of the environment and the stress from bile salts. The three types of microbeads provide probiotic protection in the following order: HPOB > CBRB > MCTB, with the probiotic counts after simulated gastric digestion being 8.06, 7.67, and 5.58 log CFU/g, respectively. MCTBs showed a reduction in probiotic viability of over 3 log CFU/g during gastric digestion and were left with only 4.65 log CFU/g after intestinal digestion. Although the gelatin shell can provide a buffered microenvironment for probiotics, enhancing their tolerance to gastric acid and bile salts, the high water-solubility of the shell leads to rapid dissolution once exposed to the digestive fluid. As a result, the MCT disintegrates quickly, causing probiotics to be directly exposed to the simulated gastric fluid and resulting in a high mortality rate, as shown in the fluorescence microscopy images (Figure 4), where no significant red fluorescence was observed in the MCTBs during oral digestion, but a large increase in red fluorescence was seen after gastric digestion. As the melting point of the lipids increased, the CBRBs and HPOBs showed better gastrointestinal protection for probiotics, with the HPOBs maintaining a viability of 8.06 CFU/g after gastric digestion, only decreasing by approximately 1 log CFU/g and still exceeding 7.5 log CFU/g after simulated intestinal digestion. This better protective effect may be due to the clumping of solid fats forming a natural barrier, reducing the direct contact between H^+^ ions and probiotics.

Therefore, considering the loss of *Lactobacillus plantarum*, the microbeads should contain a higher initial count of viable cells or use lipid matrices with higher melting points as the core to release an adequate count of cells into the intestines.

### 3.4. Storage Stability of Probiotics Loaded in Microbeads

**Figure 5:**
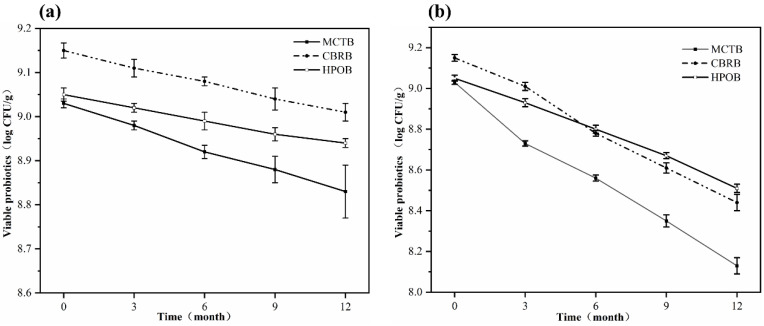
Viable counts of probiotics in microbeads during storage at 4 °C (**a**) and 25 °C (**b**).

It is well known that probiotics must maintain a certain count to exert their health benefits. Therefore, microbeads should not only ensure the survival of probiotics during the preparation process but also maintain their viability during storage. But probiotics are susceptible to external environmental factors (water activity, temperature, oxygen, etc.) during long-term storage, which can lead to a decline in their viability within the shelf life [41]. To explore the protective effects of lipid matrices with different melting points on probiotics encapsulated in microbeads, the count of viable cells during long-term storage at 4 °C and 25 °C was determined, with the results shown in Figure 5. During storage at 4 °C, all the probiotic microbeads were able to maintain a high viability of probiotics. *Lactobacillus plantarum* P9 did not show a rapid decline in the count of viable cells throughout the storage period, and it remained above 8.80 log CFU/g (Figure 5a). After 12 months, the survival rates of probiotics in the MCTBs, CBRBs, and HPOBs were all above 50%, with viable counts of 8.84 ± 0.16 log CFU/g, 9.02 ± 0.02 log CFU/g, and 8.90 ± 0.11 log CFU/g, respectively. The microbeads could maintain high probiotic viability for several reasons. (a) The low temperature slows down the metabolic rate of the cells, reduces the rate of detrimental chemical reactions, and accordingly, slows down the rate of reduction in probiotic activity [42,43]. (b) The gelatin shell layer has good gas barrier properties, which can prevent the reduction in the count of viable cells caused by the damaging effects of exposure to oxygen, and the abundant proteins in gelatin also have the capacity to enhance the survival of probiotic cells by scavenging free radicals and providing micronutrients (such as peptides and amino acids) [44]. (c) The lipid inner layer of the beads provides low water activity and high fat content, which helps to maintain high probiotic activity during storage.

In contrast to storage at 4 °C, when stored at 25 °C (Figure 5b), the rate of cell activation and basal metabolism is higher, and the rate of moisture and oxygen penetration into the microbeads from the environment is also accelerated [45], which intensifies the death of the probiotics within the microbeads. Even so, the probiotic microbeads still maintained a high count of probiotics (>8.0 log CFU/g) within 12 months. After 12 months, the viable counts of the three types of microbeads were 8.01 ± 0.04 log CFU/g, 8.44 ± 0.04 log CFU/g, and 8.51 ± 0.02 log CFU/g, respectively. This indicates that the shelf life of the microbeads can reach up to 12 months, far exceeding that of some common probiotic carriers, such as fermented dairy products. In addition, compared to before storage, the count of probiotics in the three types of beads decreased by 1.16 log CFU/g, 0.71 log CFU/g, and 0.58 log CFU/g, respectively. This suggests that solid lipids with a higher melting point have better storage stability compared to liquid fats.

Laličić-Petronijević et al. incorporated *Lactobacillus acidophilus* into dark chocolate and assessed the viability of the probiotics after storage at 4 °C and 20 °C for a period of 180 days [46]. Their findings revealed exceptionally high probiotic survival rates, with 8.49 log CFU/g and 7.77 log CFU/g, respectively. The researchers attributed this high level of probiotic viability to the anaerobic solid matrix provided by the chocolate, which appears to offer a protective environment for the probiotics. However, due to the large surface area of the chocolate, some bacterial cells might be exposed to the deleterious effects of oxygen, leading to a reduction in the count of viable cells. In contrast, the probiotic microbeads produced in this study, with their shell–core structure, avoid the shortcomings of simple lipid encapsulation and demonstrate improved probiotic stability, maintaining their functionality over long-term storage at room temperature.

Thus, the encapsulation of probiotics within microbeads allows for the creation of an environment conducive to the extended preservation and viability of the probiotics. Additionally, lipids with higher melting points provide superior protection for the probiotics during storage compared to their liquid counterparts. In the preparation of probiotic microbeads, lipids with a higher melting point can be selected as core materials to improve their storage stability, and high temperatures should be avoided as much as possible during storage.

## 4. Conclusions

This study successfully encapsulated an important probiotic (*Lactobacillus plantarum* P9) in probiotic microbeads with varying lipid melting points using high-precision concentric drop formation technology. The textural properties, preparation process, in vitro digestion, and storage stability of the microbeads are closely related to the melting point of the core lipids. Texture tests indicate that the microbeads prepared by high-precision concentric drop formation technology have good mechanical strength, and the increase in the lipid melting point is not the only factor determining the hardness of the microbeads. Viable probiotic counts show that the bacterial microbeads can maintain high cell viability during the preparation process, which is attributed to the gentle processing temperatures. Lipids with higher melting points (CBR, HPO) provide better protection for probiotics during simulated digestion and storage. Among them, HPO shows an increase of over 3.0 log CFU/g in probiotics compared to MCT during the simulated digestion process. The microbeads have a low moisture content (<4%), enabling them to maintain bacterial viability over a long period. Without changing the storage temperature, high-melting-point lipids can reduce the death rate of probiotics, maintaining their viability at room temperature for up to 12 months. This study can offer novel ideas into the design of probiotic encapsulation systems aimed at gastrointestinal protection and storage stability. Future research could focus on enhancing the performance of these microbeads by developing diverse shell materials or employing multilayer encapsulation structures, thereby ensuring the viability of the encapsulated probiotics under more stringent conditions.

## Figures and Tables

**Table 1:** Basic characteristics of microbead samples.

Samples	Wight (mg)	Diameter (mm)	Moisture Content (%)	Hardness (g)	Springiness (%)
**MCTB**	112.6 ± 1.4 ^a^	6.01 ± 0.12 ^a^	3.67 ± 0.23 ^a^	319.65 ± 35.43 ^b^	67.7 ± 5.2 ^a^
**CBRB**	111.8 ± 0.8 ^a^	6.00 ± 0.08 ^a^	3.52 ± 0.19 ^a^	623.54 ± 77.73 ^a^	43.3 ± 8.0 ^b^
**HPOB**	112.2 ± 1.0 ^a^	6.01 ± 0.09 ^a^	3.61 ± 0.33 ^a^	711.41 ± 49.57 ^a^	34.0 ± 5.5 ^b^

Means with different lowercase letters within a column demonstrate significant differences (*p* < 0.05).

## Data Availability

The original contributions presented in the study are included in the article, further inquiries can be directed to the corresponding author.
